# Kawasaki disease complicated by peripheral artery thrombosis: a case report and literature review

**DOI:** 10.1186/s12969-022-00738-y

**Published:** 2022-09-05

**Authors:** Nanjun Zhang, Li Yu, Zhongxian Xiong, Yimin Hua, Hongyu Duan, Lina Qiao, Kaiyu Zhou, Chuan Wang

**Affiliations:** 1 Department of Pediatric Cardiology, West China Second University Hospital, Sichuan University, Chengdu, Sichuan China; 2 West China Medical School of Sichuan University, Chengdu, Sichuan China; 3 The Cardiac Development And Early Intervention Unit, West China Institute of Women and Children’s Health, West China Second University Hospital, Sichuan University, Chengdu, Sichuan China; 4 Key Laboratory of Birth Defects and Related Diseases of Women and Children of Ministry of Education, Department of Pediatrics, West China Second University Hospital, No. 20, Section 3, South Renmin Road, Chengdu, 610041 Sichuan China; 5 Key Laboratory of Development and Diseases of Women and Children of Sichuan Province, West China Second University Hospital, Sichuan University, Chengdu, Sichuan China; 6 Department of Pediatrics, Second People’s Hospital of Liangshan Yi Autonomous Prefecture, Xichang, Sichuan China

**Keywords:** Prophylactic anticoagulation therapy, IVIG, Refractory KD, Peripheral artery thrombosis, Peripheral artery embolism, Peripheral gangrene

## Abstract

**Background:**

Peripheral gangrene is rarely documented as a possible complication of Kawasaki disease (KD). There are many causes of peripheral gangrene, and the common cause is in situ thrombosis or embolism. Most cases are reported to have regrettable outcomes (amputation or necrotic shedding). Herein, we report the successful management of KD complicated by peripheral artery thrombosis in an older Chinese boy, and a review of all cases of peripheral gangrene in KD in the literature.

**Case presentation:**

We found that most of the children with this complication were under 1 year old, had a heavy inflammatory response combined with the use of cortisol and immunoglobulin, and most children had coronary artery lesions. In addition, Peripheral gangrene mainly occurred in the subacute or chronic stage, and the prognosis is poor.

**Conclusions:**

In the presence of high risk factors, we consider it is necessary to monitor coagulation function and administer prophylactic anticoagulation therapy. When peripheral artery thrombosis or embolism occur, heparin and prostaglandins can be used for treatment.

## Background

Kawasaki disease (KD) is an acute systemic vasculitis of unknown origin that predominantly affects children aged 6 months to 5 years. In addition to the classic clinical features, KD can also affect multiple organs and tissues. The involvement of the pulmonary, gastrointestinal, neurological, and urinary systems has been well recognized and reported in patients with KD. In addition, aneurysms with or without extracardiac muscular arteries, including the brachial, common iliac, internal iliac, abdominal aortic, subclavian, and femoral arteries, have been recently uncovered in KD patients [1, 2].

However, peripheral gangrene has rarely been documented as a possible complication of KD. To the best of our knowledge, 25 cases have been reported in the literature. Almost all of these patients were younger than 1 year, and most were associated with coronary artery aneurysms. No similar cases have been described in the Chinese population. Most importantly, because most cases are reported with regrettable outcomes (amputation or necrotic shedding), it is of great importance and clinical significance to gain a better understanding of this rare complication in KD. Early identification and timely treatment of KD complicated by peripheral gangrene can greatly improve the prognosis and reverse the occurrence of adverse outcomes; however, little is known about this disorder in KD.

Herein, the successful management of peripheral artery thrombosis complicated by KD in an older Chinese boy was reported, and all cases of peripheral gangrene in KD in the literature were reviewed, aiming to improve the awareness and recognition of pediatricians and to share experiences regarding the management strategies for this rare complication in KD.

## Case presentation

A boy aged 4 years and 9 months was admitted to a local hospital with persistent fever for 1 day (June 4, 2021). Two days before the onset of fever, a painful lump appeared on the right side of his neck. On the second day of the disease, the child had fever with a temperature of 39 °C, and enlarged lymph nodes 2 cm × 2 cm in size, palpable in the right neck. The results of blood cell examination of the local hospital indicated a significant increase in leukocytes (leukocytes 19.25 × 10^9/L, normal range 4.03–11.09 × 10^9/L; 88% neutrophils; 6.5% lymphocytes), a decrease in hemoglobin (108 g/L, normal range 108–144 g/L), normal platelet count (385 × 10^9/L, normal range 128–420 × 10^9/L), and elevated C-reactive protein (CRP 87 mg/L, normal range 0–8 mg/L). The erythrocyte sedimentation rate was accelerated (ESR 71 mm/h, normal range, < 25 mm/h). Ultrasonography of the neck revealed enlargement of lymph nodes on both sides. The local hospital thought that the fever was caused by cervical lymphadenitis and gave the child amoxicillin-clavulanate potassium treatment (dosage and time of use were unknown). However, the child still had a persistent high fever, and new symptoms appeared on the fifth day of the disease. The patient developed a full-body congestive rash, bilateral bulbar conjunctival injection without exudate, flushed and chapped lips, swollen extremities, mild abdominal pain, and vomiting. These are the typical symptoms of KD. Because the patient had refractory fever for more than 5 days, the anti-infection treatment effect was poor, and five of the current clinical symptoms met the criteria of Kawasaki disease. Combined with white blood cells, CRP was elevated, and he was quickly diagnosed with Kawasaki disease. The local hospital quickly perfected other tests. Laboratory findings showed a significant elevation of liver enzymes (ALT 229 U/L, normal range < 49 U/L; AST 145 U/L, normal range < 40 U/L), myocardial injury with elevated brain natriuretic peptide (BNP) of 3050 pg/mL (normal range 0–100 pg/mL) and cardiac troponin T (cTnT) of 13.74 pg/mL (normal range 0–0.06μg/L). Simultaneously, color ultrasound of the abdomen showed enlargement of the liver, CT of the abdomen showed enlargement of the gallbladder, and echocardiography showed no coronary dilation. The local hospital adjusted the treatment regimen and administered aspirin (50 mg/kg, orally), IVIG (2 g/kg, intravenous drip), and antiemetic and liver-protecting medicines (specific drugs are not available). However, the treatment not effective. The vomiting and abdominal pain subsided, but the child still had a persistent high fever (after 48 h of immunoglobulin administration), and blood culture was negative. Therefore, the patient was administered a second dose of immunoglobulin (2 g/kg, intravenous drip).

On the 9th day of the illness (48 h after the second dose of immunoglobulin), the child was transferred to our hospital due to persistent high fever. Physical examination after admission showed persistent high fever, bilateral cervical lymph node enlargement, bilateral bulbar conjunctival injection without exudate, stiff and swollen extremities, warm limbs, normal arterial pulsation, and capillary filling time < 2 s. Laboratory blood tests indicated an increase in leukocytes (21.7 × 10^9/L, 92.9% neutrophils, 5.2% lymphocytes), decreased hemoglobin (81 g/L), increased CRP (194.3 mg/L), increased ESR(71 mm/h), and elevated BNP (1136.25 pg/mL). Liver function and myocardial injury index returned to normal. The electrocardiogram (ECG) was normal, and echocardiography suggested normal coronary artery (LCA = 2.9 mm, Z-score = 1.81; LAD = 2.1 mm, Z-score = 0.76; LCX = 1.9 mm, Z-score = 0.75; RCA = 2.4 mm, Z-score = 1.34). The possibility of multisystem inflammatory syndrome in children (MIS-C) [3, 4] was also excluded since two novel coronavirus nucleic acid tests outside the hospital and in our hospital were negative.

The boy was immediately administered intravenous methylprednisolone (20 mg/kg/day for 5 days) and a third dose of immunoglobulin (2 g/kg). On the 14th day of illness, the child’s temperature returned to normal. We reexamined the child’s echocardiography, which indicated left ventricular enlargement (LV = 38 mm; no dilation of left and right coronary arteries, LCA = 2.8 mm, Z-score = 1.55; LAD = 2.1 mm, Z-score = 0.76; LCX = 1.9 mm, Z-score = 0.75; RCA = 2.4 mm, Z-score = 1.34). The patient seemed to be doing well, so we gradually cut back on methylprednisolone. Unfortunately, on day 19 of the disease, the boy developed pain in his right index finger, cyanosis and desquamation of the end of the index finger, decreased temperature, and decreased palpation of the arterial pulse. Laboratory examination of disseminated intravascular coagulation (DIC) indicated blood hypercoagulability: decreased fibrinogen, increased fibrinogen degradation product, and D-dimer (Fg 50 mg/dL, normal range, 200–400 mg/dL; FDPs 38.10 μg/mL, normal range < 5μg/mL; DDI 12.02 mg/L FEU; normal range < 0.55 mg/L). Apparently, the patient had an arterial thrombosis in his right finger. Because the blood glucose of the fingertips was measured on the left hand, we ruled out thrombosis caused by an external puncture injury. At the same time, we performed a right upper limb artery ultrasound examination and echocardiography, and no obvious embolism or thrombosis was found. By reviewing the relevant literature, we found that 25 patients with KD presented with complications of peripheral gangrene [5–24], Therefore we believe that this peripheral thrombosis was related to KD.

**Fig. 1:**
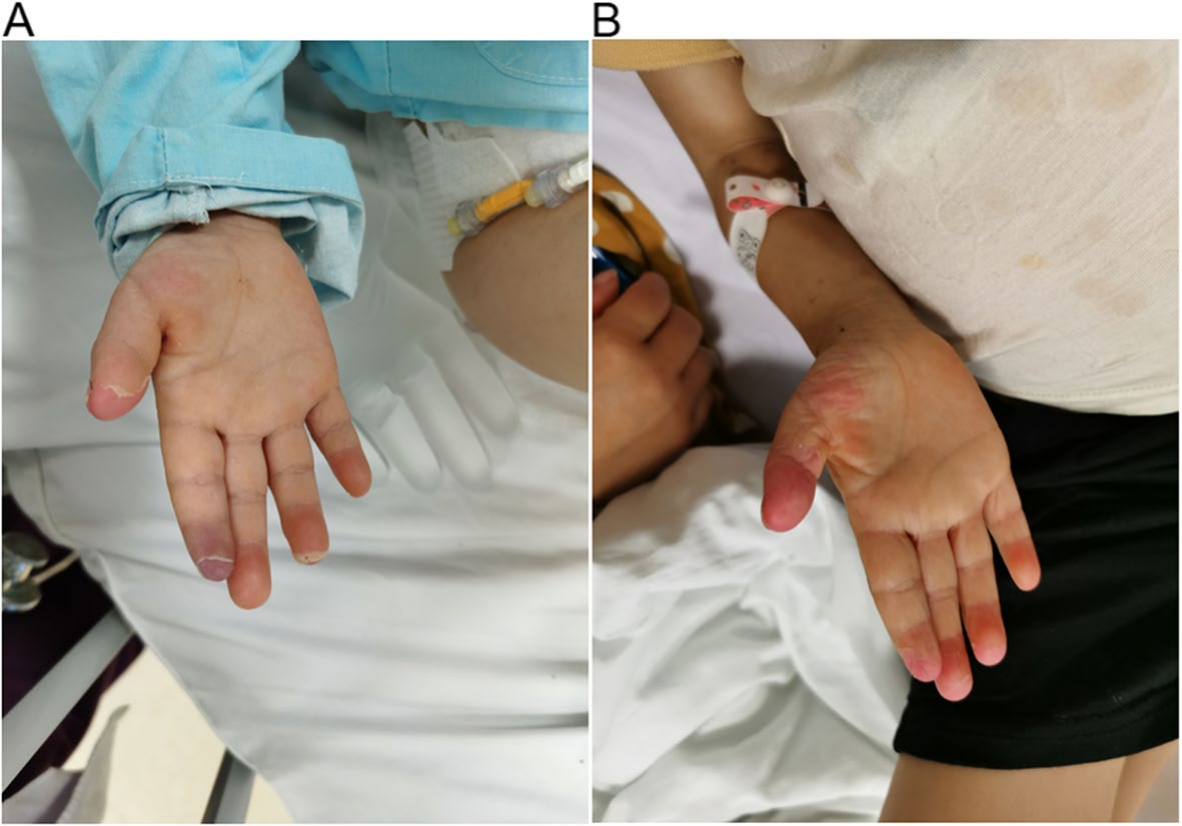
**A** On day 19 of onset, the boy presented with pain in the right index finger, cyanosis and desquamation of the end of the index finger, decreased local skin temperature, and a diminished arterial pulse on palpation. This alerted us to the complication of KD with peripheral arterial thrombosis. **B** On day 23 of onset, perfusion of the index finger limb gradually returned, and the skin of the limb was red in color

Within 4 h of peripheral arterial thrombosis discovery in the child, we successively administered urokinase (100,000 U urokinase was given three times intravenously, 30 min apart), low molecular weight heparin calcium (100 U/kg, intravenously for 5 days) and alprostadil (0.5 ng/kg/min intravenously for 5 days) for anticoagulant thrombolytic therapy. In the course of treatment, we continuously evaluated the blood supply to the right finger, avoided fingertip blood glucose measurement, and monitored coagulation function. Fortunately, perfusion of the index finger extremity was gradually restored on the 23rd day of the disease, the skin color of the extremity was ruddy, the temperature returned to normal, and the coagulation function gradually returned to normal (Fig. 1).

On the 25th day of the disease, the patient was discharged after oral aspirin administration (5 mg/kg per day). After discharge, his regular follow-up echocardiography and ECG findings were normal, and the function of his right finger was restored without sequelae. His condition was improving positively.

## Discussion

Peripheral arterial thrombosis is rare in patients with KD. We report the first case of refractory KD complicated by a peripheral artery thrombosis in a Chinese population. By reviewing the literature on 25 cases of KD complicated by peripheral gangrene, we discuss possible reasons, prevention, and treatment strategies. Based on our experience and previous information, we hope to improve peers’ understanding of KD complications and provide insights on prevention and treatment.

We compiled the clinical data of 25 children with Kawasaki disease complicated by peripheral gangrene, and we found some noteworthy phenomena: among the 25 patients (Table 1), the age distribution was mainly concentrated in infants under 1 year old (21 cases, 84%, range 1 month to 1 years, including 11 boys and 10 girls), and four children were older than 1 year (7 years, 4 years, 3.5 years, and 14 months). The mean time to onset of KD complicated by peripheral gangrene was 16.1 days (range 5th to 41stdays, 2 data were unavailable). This is different from what we thought, which occurs mainly in the subacute phase. Additionally, in terms of complications, most patients had coronary artery lesions (5 cases of dilatation and 18 cases of coronary aneurysm) and dilatation of other arteries in some cases. In terms of treatment methods, immunoglobulin use was 21/24 cases (of which multiple immunoglobulin use was 9/21 cases, some data were unavailable), most children showed IVIG resistance (16/21 cases, data were unavailable in some cases), cortisol use was 15/24 cases (some data were unavailable), and immunoglobulin combined with cortisol hormone use was 15/24 cases (some data were unavailable). In addition, the current treatment for Kawasaki disease complicated by peripheral gangrene is mainly anticoagulation, thrombolysis, and vasodilator use; of these patients, 11/21 (some data not available) remained gangrenous after treatment (eight amputations, three partial amputations, or detachments) and 13/21 recovered or improved after treatment (nine with no sequelae, three with partial recovery, and one with sequelae). Of these, 19/21 used anticoagulants (including heparin and warfarin; 10/19 improved or recovered after treatment; 9/19 amputation, partial amputation, or detachment), 6/21 used vasodilators (including nitroglycerin and prostaglandins; 5/6 improved or recovered after treatment; 1/6 amputation, partial amputation, or detachment), and 3/21 thrombolytic therapy (including t-PA and urokinase; 1/3 improvement or recovery after treatment; 2/3 amputation, partial amputation, or detachment). Anticoagulation and thrombolytic therapy were used together in 2/21 cases (1/2 improvement or recovery after treatment; 1/2 amputation, partial amputation, or detachment), and anticoagulation and vasodilators were used together in 9/21 cases (4/9 improvement or recovery after treatment; 5/9 amputation, partial amputation, or detachment).

**Table 1:** Baseline data of 25 children

References	Sex/Age (month)	Incomplete KD	Coronary artery lesions	IVIG	Corticosteroids treatment	Immunosuppressive and biological agents^b^	Peripheral gangrene site	Abnormal timing of peripheral gangrene	Recovery time from peripheral gangrene	Prognosis of peripheral gangrene	Anticoagulation, thrombolytic and other therapy (Besides aspirin)^a^
Fatemeh Tahghighi et al.	F/8	–	Dilatation	(+)/2 doses	(+)	NA	Knee	26th	56th	No significant sequelae	–
Iran Malekzadeh et al.	M/4	Incomplete	Multiple aneurysms	(+)	(+)	2	On both sides lower and right fingers	NA	–	Amputation	1
	F/6	Incomplete	Dilatation	(+)	(+)	NA	Right fingers	NA	NA	No significant sequelae	NA
	M/12	Incomplete	Multiple aneurysms	(+)/2 doses	(+)	1,4	Fingers	20th	NA	No significant sequelae	1,2
M. von Planta et al.	F/2	incomplete	Multiple aneurysms	(+)	NA	NA	Left fingers, right toes	18th	40th	No significant sequelae	1,3
Na Yeon Kim et al.	M/57d	–	Multiple aneurysms	(+)/4 doses	(+)	3	Left fingers, right toes	14th	22nd	No significant sequelae	1,2
Jai Prakash Son et al.	M/4Y	incomplete	Normal	(+)	NA	NA	Left side of the lower lip and left finger	7th	NA	No significant sequelae	1
Matthew J.O’Connor et al.	M/7 weeks	incomplete	Multiple aneurysms	(+)/2 doses	(+)	NA	Left toes and the left finger	8th	NA	NA	1,4
A. L. Durall et al.	M/1	–	Multiple aneurysms	(+)/2 doses	(+)	NA	Hands and feet	14th	–	Amputation	1,4,8
Faten Al Tasseh et al.	M/ 14	incomplete	Dilatation	(+)	(+)	NA	right fingers	9th	–	Amputation	1,3,5
Shobun Tomita et al.	F/ 2	incomplete	Multiple aneurysms and Coronary artery thrombosis	(+)/2 doses	NA	NA	Left fingers	22th	23rd, Partial improvement	Partial amputation	1,2,3,4,6,7
	M/2	incomplete	Multiple aneurysms	(+)	NA	NA	The left hand	17th	22nd, Partial improvement	Partial amputation	1,2
	F/3	–	Multiple aneurysms	(+)/3 doses	(+)	NA	left hand (at the wrist), left leg (at the knee), and	18th	–	Amputation	1
							right foot (at the ankle)				
C. Krohn et al.	F/3	Incomplete	Aneurysms	(+)	(+)	NA	The left hand and left foot	28th	–	Amputation	1,6
Mark A. Westphalen et al.	M/5	–	Aneurysms	(+)	(+)	NA	Hands	18th	28th, Most improvement	Partly leaving after-effects	3,4
Elisabeth Gomez Moyano et al.	M/4	–	Multiple aneurysms	(+)	(+)	NA	Fingers and toes	15th	–	Amputation	1,3,4
Elliot L. Ames et al.	M/4	–	Multiple aneurysms	NA	NA	NA	Hands	27th	–	Amputation	1
O H Teixeira et al.	F/7	–	Multiple aneurysms	NA	NA	NA	Fingers and toes	16th	70th, Most improvement	NA	NA
Omer Faruk Dogan et al.	F/7Y	–	Multiple aneurysms	(+)	(+)	NA	Left toes	6th	21st	No significant sequelae	1,3
Muhammad Mohsin et al.	F/10	–	Dilatation	(+)	NA	NA	Left big toe	7th	10th, Most improvement	No significant sequelae	1,4
J.-S. Chang et al.	M/8.5	–	Dilatation and Aneurysms	(+)	NA	NA	Fingers and toes	7th	23th, Most improvement	Partial amputation	1
J L Brenner et al.	F/3	Incomplete	Dilatation and Aneurysms	NA	NA	NA	Right toes	41th	–	Amputation	NA
	M/5	–	Multiple aneurysms	(+)/3 doses	(+)	NA	Big toe	28th	NA	NA	1,2,4,6
Garrido-García LM, et al.	F/45d	NA	NA	NA	NA	NA	Right toes	NA	NA	NA	NA
Madan D et al.	M/3.5Y	Incomplete	Dilatation	(+)/2 doses	(+)	4	Both hands and feet and face	5th	NA	No significant sequelae	1

^a^1. Heparin, 2. Warfarin, 3. Prostaglandin, 4. Nitroglycerin, 5. Fresh frozen plasma, 6. tissue type PA(t-PA), 7. Urokinase, 8. antithrombin III

^b^1. Azathioprine; 2. Cyclophosphamide; 3. Methotrexate; 4. Infliximab

A number of mechanisms have been proposed for the potential mechanisms of peripheral gangrene complicating Kawasaki disease, although they are not fully understood. First, the inflammatory response is associated with a hypercoagulable state of the blood. The immune inflammatory response in children with Kawasaki disease can lead to the destruction of vascular endothelial cells, followed by a decrease in platelets and activation of the coagulation system. This leads to the formation of blood clots. Most children with KD suffering from peripheral gangrene have a strong immune inflammatory response, as evidenced by the development of vascular complications, young age (< 1 year of age), immunoglobulin resistance phenomena, the need for cortisol hormones or immunosuppressants, and the presence of coronary aneurysms or other peripheral arterial damage. However, this is not absolute, and we also found that children with Kawasaki disease at older ages and without coronary artery dilation may also have peripheral gangrene complications (as in the case we reported and in a previous child with Kawasaki disease at age 7 years without coronary artery dilation), which also reminds us that older children with Kawasaki disease without coronary artery dilation, deserve to be examined. In addition, several previous studies have reported the presence of a hypercoagulable blood state in patients with KD in the acute phase, with D-dimer being associated with CRP [25–32]. The significant elevation of CRP in our patient also suggests a high risk factor. However, this is also somewhat controversial, as we found that the mean time to the onset of KD complicated by peripheral gangrene was 16.1 days (range 5th–41st days, 2 data were unavailable). Contrary to our hypothesis, it occurred mainly in the subacute phase. This may be a continuation of the rapid inflammatory response in the acute phase, but it may also be a delay in the therapeutic window owing to the difficulty of early recognition and the paucity of information available for preventive decision-making. Second, treatment with some medications seems to contribute to the development of this complication to some extent. The most common treatment for children with Kawasaki disease with intense inflammation is the use of immunoglobulins and hormones, but thrombotic complications are well-known complications of immunoglobulin therapy and may be related to increased blood viscosity, the procoagulant activity of the immunoglobulins, and the immunoglobulin-induced arterial vasospasm [33]. The use of cortisol hormones is also known to increase the viscosity of blood and promote thrombosis. The almost unavoidable use of immunoglobulins and hormones, even multiple times, in previous case reports and in our case, reminds us, on the other hand, that treatment should be accompanied by great vigilance for the development of arterial thrombosis. Third, spasms of the peripheral arteries may also play a role. The stimulation of blood vessels by drugs, spasm of peripheral arteries due to inflammation, and puncture of peripheral arteries during treatment (such as fingertip blood glucose monitoring and arterial blood gas analysis) may lead to peripheral artery spasm, thus causing a change in hemodynamics and the occurrence of peripheral artery thrombosis. Fourth, the research of Huang MY et al. [34] showed that the capillary morphology was abnormal when compared to controls in KD, with a larger diameter of the arterial and venous limbs, a higher intercapillary distance and a decrease in the loop numbers. Significantly decreased capillary blood cell velocity was noted in afebrile phase. The deceleration of the flow velocity of the extremities may become a high risk factor for thrombosis in KD and contribute to the peripheral artery thrombosis in our case. .

Treatment of KD complicated by peripheral gangrene or peripheral artery thrombosis is rare and not mentioned in current guidelines, and most cases are empirically treated from the perspective of arterial thrombosis, which makes us very passive in treatment plan decisions. The main treatment is anticoagulation, thrombolysis, and use of vasodilators; of these patients, 11/21 (some data were unavailable) were known to remain gangrenous after treatment (eight amputations, three partial amputations or detachments) and 13/21 recovered or improved after treatment (nine with no sequelae, three with partial recovery, and one with sequelae). Of these, 19/21 used anticoagulants (including heparin and warfarin, 10/19 improved or recovered after treatment; 9/19 amputation, partial amputation, or detachment), 6/21 used vasodilators (including nitroglycerin and prostaglandins; 5/6 improved or recovered after treatment; 1/6 amputation, partial amputation, or detachment), and 3/21 thrombolytic therapy (including t-PA and urokinase; 1/3 improvement or recovery after treatment; 2/3 amputation, partial amputation, or detachment). Anticoagulation and thrombolytic therapy were used together in 2/21 cases (1/2 improvement or recovery after treatment; 1/2 amputation, partial amputation, or detachment), and anticoagulation and vasodilators were used together in 9/21 cases (4/9 improvement or recovery after treatment; 5/9 amputation, partial amputation, or detachment). In addition, it is widely proved that hyperbaric oxygen therapy could ameliorate diseases with impaired oxygen supply due to capillary injury or vascular obstruction. For the reported 25 cases with peripheral artery thrombosis in KD, none of them received hyperbaric oxygen therapy and half of them remained gangrenous after treatment. Therefore, hyperbaric oxygen therapy may serve as an adjunct therapeutic option for peripheral artery thrombosis in KD if these children were unresponsive for the treatment of anticoagulation, thrombolysis, and use of vasodilators.

In summary, when a child has a high-risk factor for age less than 1 year, intense inflammatory response, multiple IVIG and cortisol hormone use, and the presence of coronary artery damage, a high degree of vigilance is needed for the development of peripheral artery thrombosis or peripheral gangrene, especially during the subacute phase of KD. However, we report a case of Kawasaki disease complicated by peripheral artery thrombosis in an older child without coronary artery damage in China, which on the other hand reminds us that an older child without coronary artery damage needs equal attention. When this KD complication occurs, early treatment with thrombolysis combined with anticoagulation seems to reduce the occurrence of adverse outcomes, and prostaglandin analogs can be used as a therapeutic option.

## Conclusions

KD complicated by peripheral artery thrombosis is very rare, and it can lead to peripheral gangrene. The early prevention, recognition, and treatment can reduce the probability of amputation. We report the first case of an older Chinese child with KD complicated by peripheral artery thrombosis. Through this case report and literature review, we found that most children with this complication were under 1 year old, had a heavy inflammatory response combined with the use of cortisol and immunoglobulin, and most children had coronary artery lesions. In addition, peripheral gangrene mainly occurs in the subacute or chronic stage, and the prognosis is poor. Therefore, in the presence of these high-risk factors, it is necessary to monitor coagulation function and administer prophylactic anticoagulation therapy. Heparin and prostaglandins can be used for treatment of peripheral artery thrombosis.

## Data Availability

The original contributions presented in the study are included in the article/supplementary material, further inquiries can be directed to the corresponding authors.

## Abbreviations

KD: Kawasaki disease
IVIG: Intravenous immunoglobulin
ECG: Electrocardiogram
DIC: Disseminated intravascular coagulation
